# Mosquito Brigades, and How to Organize Them

**Published:** 1903-04

**Authors:** 


Mosquito Brigades, and How to Organize Them.—By Ronald Ross, F. R. C., D. P. H., F. R. S., Lecturer in Tropical Medicine, Liverpool School of Tropical Medicine, Major Indian Medical Service, Retired. 100 pages, price not given. New York, Longmans, Green & Co. London, George Phillip & Son. 1902.

The purpose of the book is to show how the disease carrying breeds of mosquitoes can be completely annihilated. The author considers screens are only for the rich. The common herd cannot afford them and must be the chief victims of diseases. Get rid of the pests is the thing to do. This is done, according to the author, by the use of petroleum and other destructive culicides, and by the filling up of all pools and stagnant places where the mosquito is propagated. The book is interesting, and if time shows that the mosquito theory is the correct one, the work will have, and should have, a very large sale. T. J. B.

